# Evaluation of Two Portable Hyperspectral-Sensor-Based Instruments to Predict Key Soil Properties in Canadian Soils †

**DOI:** 10.3390/s22072556

**Published:** 2022-03-26

**Authors:** Nandkishor M. Dhawale, Viacheslav I. Adamchuk, Shiv O. Prasher, Raphael A. Viscarra Rossel, Ashraf A. Ismail

**Affiliations:** 1 Department of Bioresource Engineering, McGill University, Sainte-Anne-de-Bellevue, QC H9X 3V9, Canada; shiv.prasher@mcgill.ca; 2 Department of Soil and Landscape Science, Faculty of Science and Engineering, School of Molecular and Life Sciences, Curtin University, G.P.O. Box U1987, Perth, WA 6845, Australia; r.viscarra-rossel@curtin.edu.au; 3 Department of Food Science and Agricultural Chemistry, McGill University, Sainte-Anne-de-Bellevue, QC H9X 3V9, Canada; ashraf.ismail@mcgill.ca

**Keywords:** chemometrics, hyperspectral sensor instruments, on-the-spot soil analyzers, portable spectrophotometers, proximal soil sensing, sensors, soil spectroscopy, visible and near-infrared spectroscopy, mid-infrared spectroscopy

## Abstract

In contrast with classic bench-top hyperspectral (multispectral)-sensor-based instruments (spectrophotometers), the portable ones are rugged, relatively inexpensive, and simple to use; therefore, they are suitable for field implementation to more closely examine various soil properties on the spot. The purpose of this study was to evaluate two portable spectrophotometers to predict key soil properties such as texture and soil organic carbon (SOC) in 282 soil samples collected from proportional fields in four Canadian provinces. Of the two instruments, one was the first of its kind (prototype) and was a mid-infrared (mid-IR) spectrophotometer operating between ~5500 and ~11,000 nm. The other instrument was a readily available dual-type spectrophotometer having a spectral range in both visible (vis) and near-infrared (NIR) regions with wavelengths ranging between ~400 and ~2220 nm. A large number of soil samples (*n* = 282) were used to represent a wide variety of soil textures, from clay loam to sandy soils, with a considerable range of SOC. These samples were subjected to routine laboratory soil analysis before both spectrophotometers were used to collect diffuse reflectance spectroscopy (DRS) measurements. After data collection, the mid-IR and vis-NIR spectra were randomly divided into calibration (70%) and validation (30%) sets. Partial least squares regression (PLSR) was used with leave one out cross-validation techniques to derive the spectral calibrations to predict SOC, sand, and clay content. The performances of the calibration models were reevaluated on the validation set. It was found that sand content can be predicted more accurately using the portable mid-IR spectrophotometer and clay content is better predicted using the readily available dual-type vis-NIR spectrophotometer. The coefficients of determination (R^2^) and root mean squared error (RMSE) were determined to be most favorable for clay (0.82 and 78 g kg^−1^) and sand (0.82 and 103 g kg^−1^), respectively. The ability to predict SOC content precisely was not particularly good for the dataset of soils used in this study with an R^2^ and RMSE of 0.54 and 4.1 g kg^−1^. The tested method demonstrated that both portable mid-IR and vis-NIR spectrophotometers were comparable in predicting soil texture on a large soil dataset collected from agricultural fields in four Canadian provinces.

## 1. Introduction

Several soil properties/attributes can be assessed rapidly and simultaneously, both in the laboratory and directly in the field, using hyperspectral sensor instruments that are based on diffuse reflectance spectroscopy (DRS). Numerous studies conducted in the past have shown the fruitful use of vis and NIR spectrophotometers to determine important soil attributes such as clay, soil moisture, SOC, total carbon (TC), and total nitrogen (TN) simultaneously [1,2,3,4,5,6]. Using a spectrophotometric technique, the soil sample under investigation is analyzed for the absence, presence, and quantification of various physical, chemical, and biological attributes (properties) by letting it interact with ultraviolet (UV), vis, NIR, and mid-IR radiation [1]. In general, any UV, vis, NIR, and mid-IR spectrophotometer is based on the sample’s absorption of electromagnetic (EM) radiation at wavelengths in the range of 200–25,000 nm, where intense fundamental molecular frequencies related to soil components occur between wavebands 25,000 and 2500 nm [1]. Weak overtones and combinations of these fundamental vibrations due to the stretching and bending of NH, OH, and CH groups dominate the NIR (700–2500 nm) and electronic transitions in the vis (400–700 nm) portions of the electromagnetic (EM) spectrum [1].

Based on the previously conducted studies by other researchers [1,7] it was also observed that the prediction of soil properties using spectral responses was direct or indirect in nature and therefore, based on this observation, the soil attributes (physical, chemical, and biological) could be classified into two types; viz., primary and secondary. For example: clay, soil water content (SWC), SOC, and TN are found to be primary properties for which predictions have direct spectral responses in the NIR range, and cation-exchange capacity (CEC), electrical conductivity (EC), enzyme activities, microbial respiration and microbial biomass, pH, potentially mineralizable N, sand, silt, specific surface area, and wet aggregate stability are the secondary properties that are NIR spectroscopic predictable because of their correlation with certain primary properties [7]. On the other hand, mid-IR spectroscopy is often reported to be more advantageous than vis and NIR [8,9,10,11,12,13,14], since it is more sensitive to both organic and inorganic phases of the soil. This suggests its wide usage in agricultural and environmental soil sensing applications. However, this is still being debated [15,16,17,18,19,20,21,22,23].

Soil analysis using classic laboratory-based bench-top mid-IR techniques was reviewed previously and the published applications of vis, NIR, and mid-IR spectroscopy in soil analysis for the determination of primary and secondary properties were summarized [1,9,18]. Table 1 summarizes a selected set of examples from the literature where quantitative predictions of sand, clay, and SOC were performed using spectral responses from both vis-NIR and mid-IR spectrophotometers. 

In our study, portable vis-NIR and mid-IR instruments were used in order to determine their efficacy when compared with the classic bench-top hyperspectral-sensor-based instruments. These portable instruments are built using semiconductor technologies and they are relatively inexpensive, robust, and simpler to use when compared to laboratory-based bench-top instruments. They are suitable for deployment in the field for proximal sensing [1,19,20,21,22,23,24,25,26,27,28,29,30,31,32,33]. Numerous studies support their use for the simultaneous assessment of primary and secondary soil properties on the go [34,35,36]. As an alternative to on-the-go technologies, on-the-spot measurements can be made where the field surface coverage does not allow for continuous engagement between soil and the moving parts of the sensor system (e.g., pasture). To help explore this method, a ruggedized agro-vehicle mountable platform carrying vis-NIR spectrophotometer instruments to the field has been developed (Veris^®^ P4000, Veris Technologies Inc., Salina, KS, USA). The main functional benefit of using the rugged P4000 system is its ability to measure vis-NIR spectra, cone index (CI), and soil electrical conductivity (EC) for soil profiles down to a 1 m depth.

In the scientific literature to date, up to four recent studies have reported the successful use of the vis-NIR instrument P4000 to predict soil texture, SOM, SOC, and plant-available phosphorus (PMehlich-3) [37,38,39,40,41,42]. In one of the studies, similar results for SOC prediction were found while using vis-NIR obtained using P4000 (in situ), versus spectra obtained using a laboratory-grade vis-NIR instrument (ex situ). In another study, PMehlich-3 was predicted using vis-NIR spectra (ex situ) obtained using P4000 compared to conventional laboratory measurements with an R^2^ of 0.85 and root mean squared error of 0.28 g kg^−1^. The other two studies reported predictions with root mean squared errors of 3.1 g kg^−1^ SOM, 66 g kg^−1^ sand, and 55 g kg^−1^ clay.

Recently, an all-terrain vehicle (ATV) mounted on-the-spot soil analyzer (OSA) was developed to deploy a suite of ion-selective electrodes (ISE) for subsurface measurements of chemical characteristics in a consistent and ergonomic manner [43]. A prototype mid-IR spectrophotometer is a further addition to the ISE which will allow for the integration of sensors based on different measurement principles [44,45]. However, applicability of this instrument to predict SOC and texture in comparison with the earlier mentioned P4000 unit needs to be assessed on many diverse soil samples [46].

Soil moisture and other environmental factors affect the soil spectra recorded under field conditions [1]. In addition, other systematic factors such as viewing angle may also affect the end results [44,47]. For example, the authors in [44] analyzed the repeatability of vis-NIR and mid-IR soil spectral data obtained using different measurement techniques and found that several wavelengths of the spectra were highly repeatable whereas several parts of the wavelengths were less repeatable as well. However, to make the comparisons less complicated, these factors can be eliminated by collecting repeated spectral measurements in ex situ conditions and later the instrument’s potential can be assessed in the field. The main aim of the study reported in this paper was to evaluate two portable hyperspectral-sensor-based instruments potentially deployable for in situ operations to predict key soil properties such as sand, clay, and SOC by testing 282 archived soil samples collected in contrasting agricultural fields across four Canadian provinces.

## 2. Materials and Methods

### 2.1. Soils

A total of 282 soil samples were collected and archived during two studies conducted from 2000 to 2007. These soil samples were taken from experimental plots in four Canadian provinces with humid soil moisture regimes [48]. Sixty-nine percent (*n* = 195) of the samples were from 43 sites located on research farms and in farmers’ fields in the major corn-growing regions of Québec [48]. The remaining thirty-one percent of soil samples (*n* = 87) were collected from trials having four to five replicates with corn production in four Canadian provinces: one site each in British Columbia and Ontario, two sites in New Brunswick, and five sites in Québec. All soil samples were the aggregates of 15 cm long cores collected before the application of fertilizer in the spring. They were all air-dried and ground manually using a mortar and pestle to pass through a 0.25 mm sieve in order to process them further using conventional laboratory analysis. SOC content for all soils was determined by the dry combustion method using a CNS-1000 (Leco Inc., St. Joseph, MI, USA) and soil texture was determined using the hydrometer method [49].

### 2.2. Spectral Data Collection

**Figure 1:**
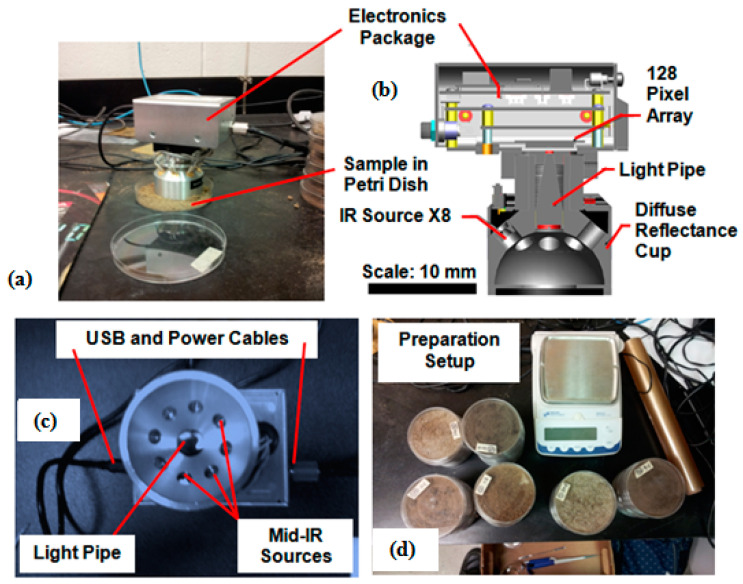
Illustration of the laboratory-configured portable mid-IR spectrophotometer (collecting measurements on soils in Petri dish (**a**), cross-sectional view (**b**), USB and power supply connections, light pipe, and sources of eight mid-IR (**c**), preparation setup (**d**)).

At first, and as illustrated in Figure 1a,b, the mid-IR spectra of soil samples were acquired using a lightweight mid-IR spectrophotometer from Wilks Enterprise Inc. (East Norwalk, CT, USA). This instrument is based on variable-filter-array (VFA) diffuse reflectance infrared Fourier transform (DRIFT) technology. In the prototype, a linear variable filter (LVF) detector was placed on the top center inside the enclosure and was mounted on a pyroelectric detector array (size = 128 pixel) above an optical window made of zinc-selenide (ZnSe). The prototype spectrophotometer consisted of eight sources of infrared light (which were modulated using a specialized electronic circuit) and was capable of maintaining a constant distance between the detector and the measured soil surface (Figure 1c). Per each scan operation, an average of eight pulsed reflectance measurements was recorded. The VFA in the prototype was configurable through hardware settings. This was useful since it helped to provide flexibility of operation in both the NIR and mid-IR ranges of the EM spectrum. In this study, the LVF was configured to obtain spectra in the short mid-IR range (5500–11,126 nm) with ~44 nm of spectral resolution.

The electronic control circuitry was enclosed in the superior part of the prototype spectrophotometer and was powered using a specially designed 12 volt DC adapter (Figure 1a–c). The prototype spectrophotometer was interfaced with a standard desktop computer through a USB 2.0 cable (Figure 1c). A special purpose spectral data acquisition (DAQ) software called C-One (Cogni-Solve, Inc., Montréal, Québec, QC, Canada) was used to acquire and record soil diffuse spectral measurements in the absorbance format. As illustrated in Figure 1a,d, the mid-IR spectral data collection process involved placing the prototype spectrophotometer over the soil sample (~10 g, filled and evenly spread in a Petri dish) of radius 15 mm and thickness of 5 mm (area ~707 mm^2^) and for ~32 s. To minimize random and systematic noise, each mid-IR spectrum was recorded as an average of 32 scans. As specified by the manufacturer, the prototype spectrophotometer was recalibrated every 5–7 samples by using standard reference material (a special purpose sheet whose surface color was a golden shade).

**Figure 2:**
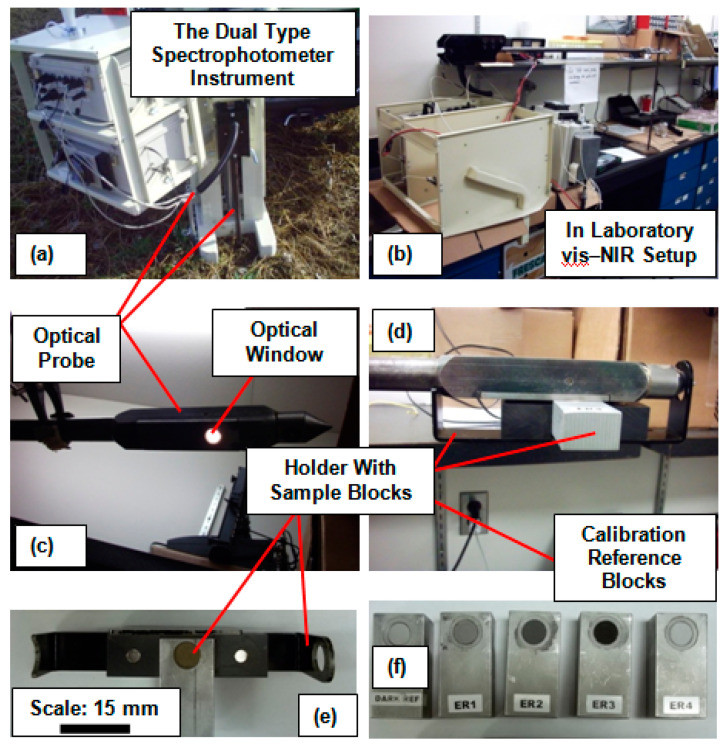
Illustration of the field and laboratory configurable portable vis-NIR spectrophotometer (transportable field setup (**a**), laboratory setup with vis-NIR probe (**b**), the optical window made out of sapphire (**c**), vis-NIR probe with sample and its holder for collection of vis-NIR spectra (**d**), sample holder attachment with sample holder filled with soil sample (**e**), and reference blocks for spectral calibrations (**f**)).

As shown in Figure 2a,b, the second hyperspectral-sensor-based instrument (part of the ruggedized P4000) was a dual kind of spectrophotometer system, because it operated in both the visible and near-infrared regions of the EM spectrum. Among the dual spectrophotometers, the first was the USB2000 (Ocean Optics Inc., Dunedin, FL, USA) which operated in the range of wavelengths between 342 and 1023 nm, with a spectral resolution of 6 nm. The other spectrometer was a mini-spectrophotometer (model no. C9914GB, Hamamatsu Photonics. K.K., Tokyo, Japan) which operated in the range of wavelengths between 1070 and 2220 nm, with a spectral resolution of 4 nm. The dual spectrophotometer was operated using its own light source and was capable of maintaining a constant gap between the fiber optic probes and the measured soil samples using a designated sapphire window (Figure 2c,d).

As illustrated in Figure 2c, the vis-NIR spectral data collection process was conducted by depositing ~1 g of soil into a specially designed sample holder (thickness of 5 mm and radius of 5 mm) and placing it in contact with an optical window of area ~79 mm^2^ (Figure 2c). The dual hyperspectral sensor instrument was optimized and calibrated by measuring the dark current followed by white reference measurements using the specially provided reference blocks (Figure 2d). The dual hyperspectral sensor instrument was calibrated at the beginning of the data collection procedure and was recalibrated every 20 samples. Each spectrum was recorded as an average of 30–32 scans, to minimize random and systematic noise. Finally, the vis-NIR soil spectra were interpolated to ~5 nm of spectral resolution, yielding a total of 380 data points (wavelengths) per spectrum.

### 2.3. Spectral Data Processing

**Figure 3:**
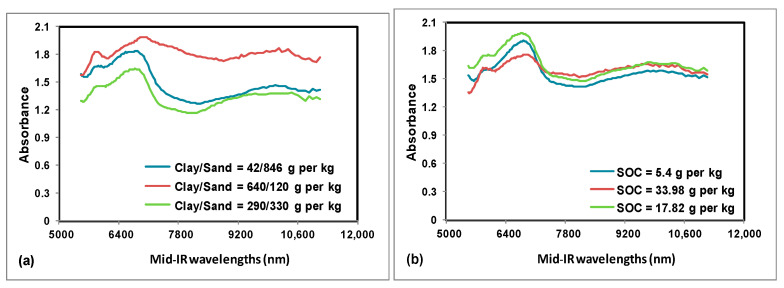
Example of portable mid-IR soil absorbance measurements recorded on selected soil samples. With varying sand and clay (**a**) and SOC content (**b**).

**Figure 4:**
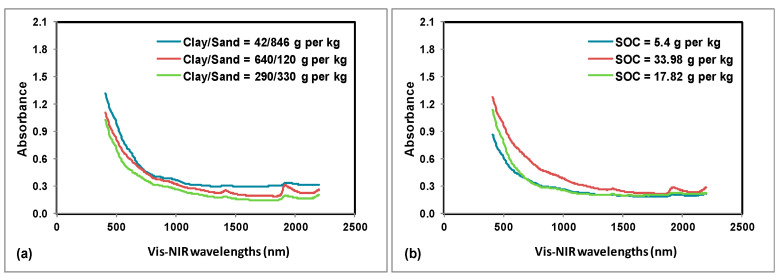
Example of portable vis-NIR soil absorbance measurements recorded on selected soil samples with varying sand and clay (**a**) and SOC content (**b**).

From both instruments, the raw spectral data points were imported into the file system of the chemometric software application called ParLeS (University of Sydney, Sydney, Australia) and preprocessed in a similar way to the method discussed in [50]. As illustrated in Figure 3, the obtained mid-IR soil absorbance spectra on varying sand, clay, and SOC contents looked different from each other and consisted of the total 128 absorbance measurements per spectra. They also had a short range with a course resolution between each wavelength. As shown in Figure 4, the obtained vis-NIR soil reflectance spectra on varying sand, clay, and SOC contents looked different from each other and exhibited a small step discontinuity between 1022 and 1071 nm, due to the transition from one spectrophotometer to the other. The noisy parts in vis-NIR spectra were filtered out by removing the noisy “edges” of each spectrum (342–409, 1014–1075, and 2206–2220 nm). Initially, these operations were performed using Microsoft Excel software application (Microsoft Inc., Redmond, WA, USA) and it produced a spectrum of 365 original data points per sample. Later, the resulting 365-point vis-NIR spectra were transformed into absorbance measurements. Finally, and wherever applicable, for both mid-IR and vis-NIR spectra, the further data preprocessing steps [44] in ParLeS [50] included: offset correction using multiplicative scattering correction (MSC) algorithm, Ref. [51] Savitzky–Golay (SG) smoothing, Ref. [52] as well as mean centering (MC). The parameters chosen for SG smoothing were first derivative of window size = 11, second derivative of window size = 11, and first derivative of window size = 9 for sand, clay, and SOC, respectively.

**Figure 5:**
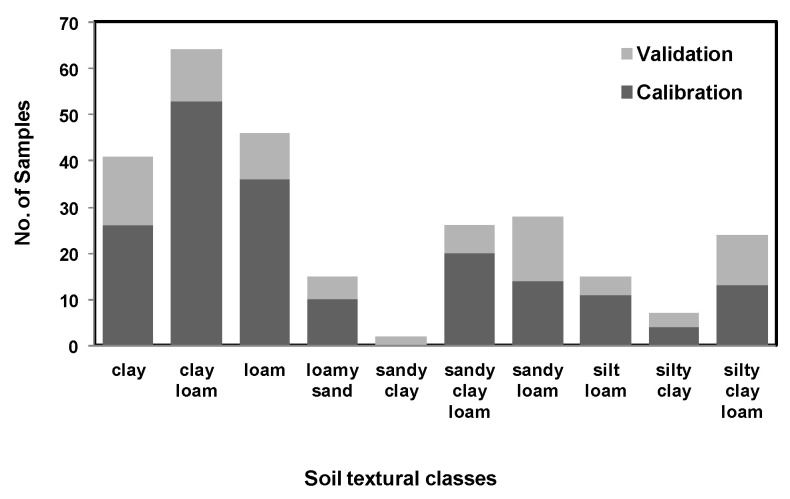
Chart (stacked histogram with two categorical variables (calibration data and validation data)) illustrating the distribution of textural soil class based on USDA [44,52].

Both mid-IR and vis-NIR spectra were randomly partitioned into two sets only once: a calibration set consisting of 70% of soil sample examples and a validation set consisting of 30% of soil sample examples. This way, care was taken so that it covers a similar range of soil property quantities between calibration and validation sets. As a result, soil examples with different textures [45,53] and SOC content were split between calibration and validation samples as shown in Figure 5. A partial least squares regression (PLSR) with leave-one-out cross-validation was used for spectral calibration against laboratory measurements. The orthogonalized PLSR-1 algorithm implemented in ParLeS was used [54]. This is a bilinear regression technique that extracts a small number of latent factors, which are a combination of the explanatory variables of reflectance or absorbance (at spectral wavelengths or wavenumbers), and uses these factors to produce a regression for the dependent variables [55,56].

The soil properties analyzed using standard laboratory procedures were common between the calibration and validation datasets for both vis-NIR and mid-IR. On both, i.e., vis-NIR and mid-IR calibration datasets, leave-one-out cross-validation was used to select the number of PLSR factors to use in each model. Later, the selected calibration models were tested on the validation dataset. The performance of all models was evaluated using the statistics: root mean squared error (RMSE), coefficient of determination (R^2^), the standard deviation of the error distributions (SDE), and the mean error (ME). The RMSE is a mix of both the ME and the SDE, where ME indicates the bias and SDE represents a random error. The equation follows:

(1) $$R^{2}=1-\frac{\mathrm{SSE}}{{\mathrm{SS}}_{\mathrm{yy}}}$$

(2) $${\mathrm{SS}}_{\mathrm{yy}}=\sum_{i=1}^{n}{\left(y_{i}-\bar{y}\right)}^{2}$$

(3) $$\mathrm{SSE}=\sum_{i=1}^{n}{\left(y_{i}-\overset{⌢}{y}\right)}^{2}$$

(4) $$\mathrm{RMSE}=\frac{1}{N}\sum_{i=1}^{N}({\overset{⌢}{y}}_{i}-y_{i}{)}^{2}$$

(5) $$\mathrm{ME}=\sum_{i=1}^{N}({\overset{⌢}{y}}_{i}-y_{i})$$

(6) $$\mathrm{SDE}=\frac{\sum_{i=1}^{N}({\overset{⌢}{y}}_{i}-y_{i}-{\mathrm{ME})}^{2}}{N-1}$$

In Equations (1)–(6), $\bar{y}$ is the mean of observation values, N is the sample size, SSE is the difference of observation from their predicted values, ${\mathrm{SS}}_{\mathrm{yy}}$ is the difference of the observation from the mean, $y_{i}$ is the laboratory-measured unknown observed value of the soil property of interest, and ${\overset{⌢}{y}}_{i}$ is the predicted value of the soil property of interest.

Further, the PLSR coefficients (betas) and variable importance for projection (VIP) [57] were used to identify the mid-IR and vis-NIR frequencies (wavelengths) used to predict sand, clay, and SOM contents in the calibration sets of the soils.

## 3. Results and Discussion

**Figure 6:**
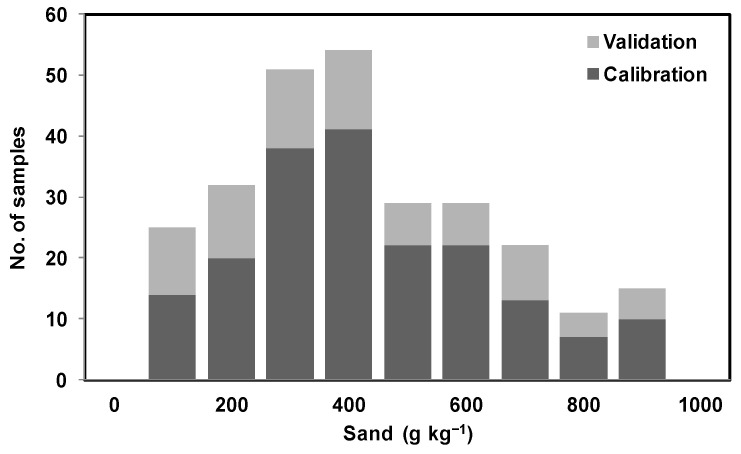
Illustration of distribution of sand content using stacked histogram with two categorical variables (calibration data and validation data).

**Figure 7:**
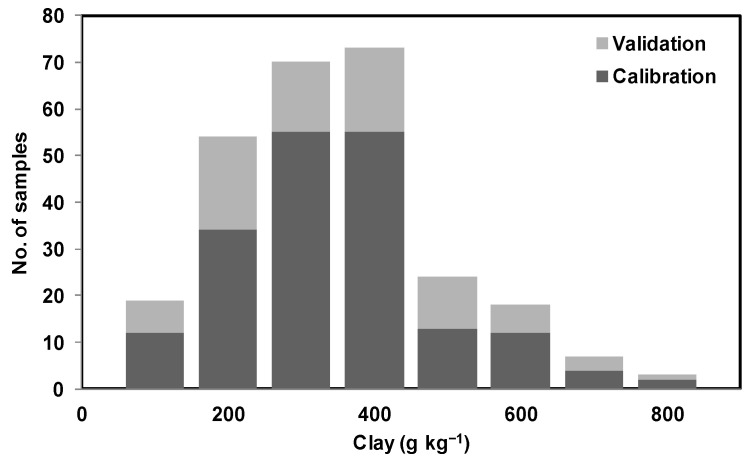
Illustration of distribution of clay content using stacked histogram with two categorical variables (calibration data and validation data).

**Figure 8:**
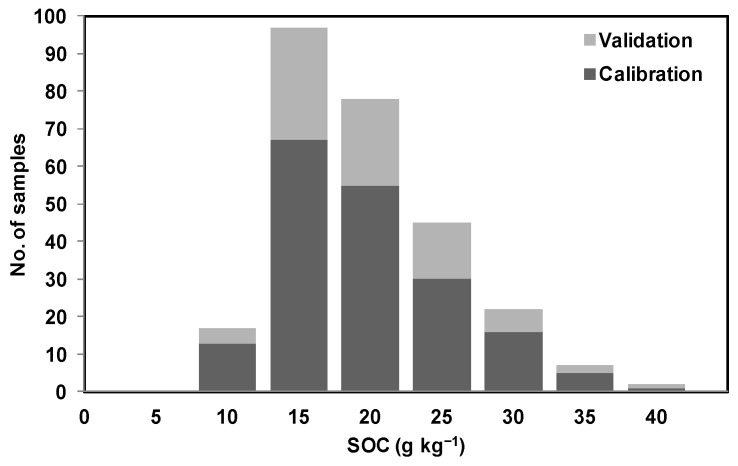
Illustration of distribution of SOC content using stacked histogram with two categorical variables (calibration data and validation data).

As shown in Table 2 and Figure 6, Figure 7 and Figure 8, sand and clay content were observed to range from 0 to 860 g kg^−1^ and 42 to 760 g kg^−1^, respectively, and a substantial range between 5 and 39 g kg^−1^ was observed in the SOC content. Among the soil spectral calibration samples obtained using mid-IR, three models were found to be appropriate (using a relatively small number of latent factors (5, 4, and 5)) in predicting sand, clay, and SOC content with R^2^ and RMSE ranging between 0.61 and 0.64 and 3.7 g kg^−1^ and 121.4 g kg^−1^ (Table 3). For the validation sets of soil spectral samples, the R^2^ and RMSE were 0.82 and 103.3 g kg^−1^ sand, 0.78 and 77.9 g kg^−1^ clay, and 0.54 and 4.1 g kg^−1^ SOC. A minor difference was observed between the obtained results using calibration and validation datasets. Moreover, small ME values indicate a good split between calibration and validation sets of soil resulting in the absence of bias.

**Figure 9:**
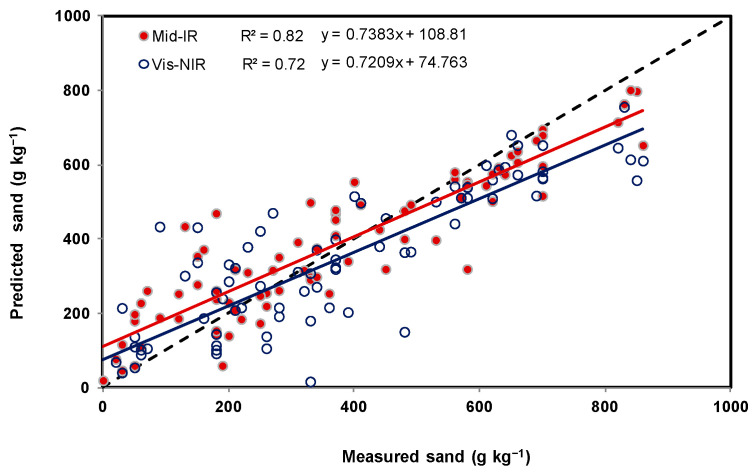
Relationships between measured and predicted sand, with dots representing validation examples in accordance with Table 3 and Table 4.

**Figure 10:**
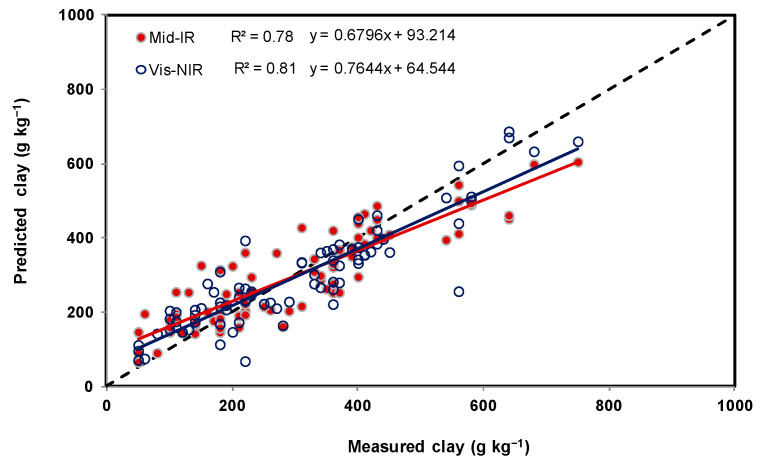
Relationships between measured and predicted clay, with dots representing validation examples in accordance with Table 3 and Table 4.

**Figure 11:**
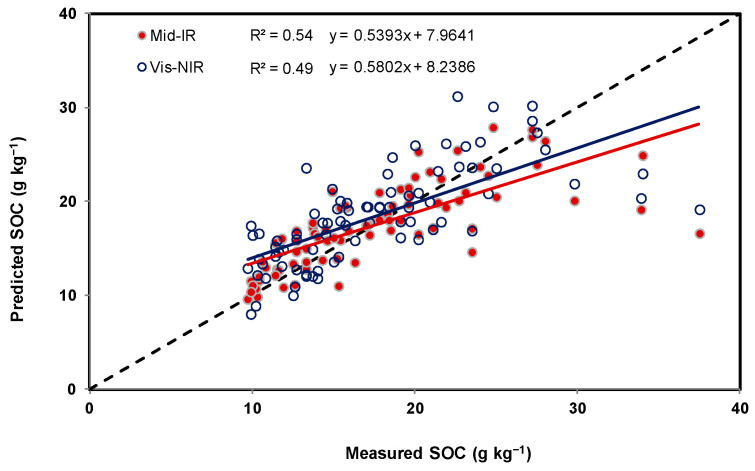
Relationships between measured and predicted SOC, with dots representing validation examples in accordance with Table 3 and Table 4.

In terms of the vis-NIR soil spectral calibration (Table 4), relatively greater numbers of latent factors (15, 7, and 12) were found to be appropriate in predicting sand and clay, with R^2^ and RMSE ranging between 0.62 and 0.79 and 3.7 g kg^−1^ and 104 g kg^−1^. For the validation set of soil, the R^2^ and RMSE were 0.74 and 127.3 g kg^−1^ sand, 0.82 and 71.7 g kg^−1^ clay, and 0.49 and 4.5 g kg^−1^ SOC. The remaining observations were similar to the mid-IR spectra. Relationships between measured and predicted sand, clay, and SOC contents, using both spectrophotometer techniques, are illustrated using Figure 9, Figure 10 and Figure 11. As indicated by the RMSE (Table 3 and Table 4), there was no observable difference in terms of the performances of both hyperspectral sensor instruments. Twice the difference indicated by ME could have been due to the difference in the exposed soil sample area or being brought in contact with the detectors of both hyperspectral sensor instrument systems, i.e., due to the fact that averaged absorbance values reported by the mid-IR detector were collected from a sample area that was −10 times greater than the sample area used during vis-NIR-based data collection.

One more observation of this study was that, while predicting sand and clay using mid-IR and clay using vis-NIR, the associated R^2^ and RMSE for validation datasets were better than the calibration datasets. This could possibly be due to the data being randomly split between calibration and validation only once, while taking care that the similar range for soil property quantities remained common between the calibration and validation datasets (Figure 5). However this possibility was not tested and could be tested in future by splitting the data many times and modeling the data using the five-fold cross-validation technique [47].

**Figure 12:**
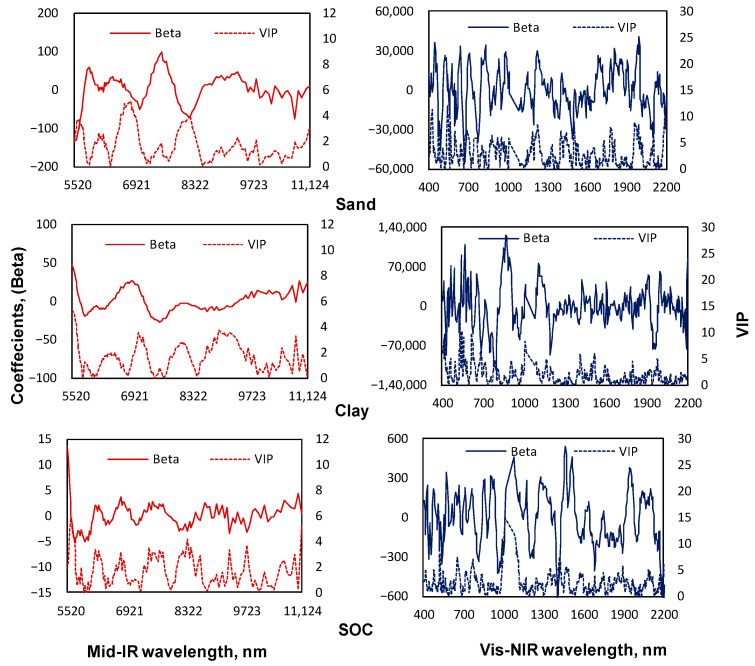
Illustration of the PLSR coefficients (betas) and variable importance of projections (VIP) index related to the prediction of sand, clay, and SOC contents in soil samples using portable mid-IR and vis-NIR spectra for calibration datasets.

Figure 12 shows the regression coefficients and the VIP. Based on the number of latent factors used for PLSR modeling (Table 3 and Table 4) and as assessed by the sizes of their negative or positive peaks and VIP peaks for mid-IR spectra, wavebands near 5609, 6818, 7642, 8279, and 10,781 nm were important for predictions of sand; near 5520, 6294, 6990, and 7601 nm for predictions of clay, and those near 5520, 5678, 5945, 6852, 9398, and 11,038 nm were most important for predictions of SOC. Similarly, for the vis-NIR spectra, wavebands near 445, 504, 533, 562, 706, 765, 827, 986, 1223, 1228, 1447, 1697, 1808, 1992, 2091, and 2193 nm were useful for predicting sand, those near 463, 539, 568, 685, 806, 852, 872, 896, 1113, 1197, 1948, 1964, and 2193 nm for predicting clay, and near 439, 539, 568, 811, 857, 911, 986, 1202, 1226, 1403, 1462, 1503, 1683, 1948, 1952, 2115, and 2186 nm for predicting SOC.

When comparing with previous literature [14,45,58,59,60,61,62,63], the above listed mid-IR wavebands and their direct or indirect association pairs are to be: 5609, 5520, and 5945 nm with, H_2_O; 6818, 6990, and 6852 nm with calcium carbonate; 9398 nm with carbohydrate; 5609 and 5678 nm with organic material; 5520 and 6294 nm with clay material; 10,781 and 11,038 nm with silicon dioxide. Whereas, the vis wavebands near 445, 504, 533, 562, and 706 nm indirectly correlate with SOC due to color and the vis-NIR waveband near 1447 nm was found to help in predicting sand and the waveband near 1948 nm was found to help in predicting clay, where at 1420 nm and 1920 nm, both wavebands are known to be associated with water adsorption.

Similar to the results reported in an earlier study [1], sand was better predicted using mid-IR and clay using vis-NIR. It was found that the hyperspectral analysis of dry, crushed, sieved, and milled soil samples can reveal clay and sand content with a standard error less than 120 g kg^−1^ and for SOC, less than 5 g kg^−1^. Because silt can be estimated using clay and sand predictions and its distribution in the dataset failed to uniformly represent the entire range (required to find an optimal PLRS model), predicting this property was omitted.

Apart from the difference in costs of the used instruments and the level of portability, there was no obvious benefit of vis-NIR system over mid-IR, or vice versa. The stated error estimates reported in this study and in earlier studies (Table 1) [1,2,3,4,5,6,7,8,9,10,11,12,13,14,19,20,21,22,23,24,25,26,27,28,29,30,31,32,33,34,39,40] are higher than those corresponding to traditional analysis, where the laboratory method has a detection limit of 20 g kg^−1^ for sand and clay (dry basis), and generally, is reproducible to within ±80 g kg^−1^, Ref. [49]. In terms of SOC, the standard error was 2.5–5 g kg^−1^, Ref. [64], which is compatible with the RMSE estimated in this study. These results do not necessarily represent the potential predictability of soil texture and SOC in situ under different soil water content and bulk density. Nevertheless, an increased number of less accurate yet unbiased measurements could significantly increase the overall accuracy of thematic soil maps [65,66,67,68,69,70,71], such as SOC and texture.

The next step of this research would be to assess the portable mid-IR and vis-NIR soil spectra obtained under field conditions and further eliminate the environmental and systematic factors [44,63] affecting both soil spectra in a similar way to [46,47,72]. In addition, a variety of calibration models (ranging from simple to complex), e.g., partial least squares regression (PLSR) [1,73,74], principal component regression (PCR) [7,75], artificial neural networks (ANN) [76], convolution neural networks (CNN) [77,78,79], support vector machines (SVM) [80], and deep learning [81,82,83,84], to name a few, could be studied.

## 4. Conclusions

Two portable spectrophotometers of the mid-IR and dual vis-NIR kind were evaluated to predict key soil properties such as SOC and texture (sand and clay) in a large dataset (*n* = 282 soil samples) collected from proportional fields in four Canadian provinces. In contrast to vis-NIR spectroscopy, the use of leave-one-out cross-validation to establish a PLSR model on mid-IR calibration samples required fewer latent factors. Otherwise, there was no significant difference between the performance indicators of the two instruments. It was found that, with relatively small differences, the use of the portable mid-IR spectrophotometer was better at predicting sand, and the use of the rugged dual-type vis-NIR spectrophotometer could predict clay better. Coefficients of determination (R^2^) were observed to be optimum for sand (0.82) and clay (0.82). The matching mean square errors were 103 g kg^−1^ and 78 g kg^−1^, respectively, which were also similar to previously reported studies. The ability to predict SOC content precisely was not particularly good for the dataset of soils used in this study, with an R^2^ and RMSE of 0.54 and 4.1 g kg^−1^, respectively. The tested method demonstrated that portable mid-IR and dual-type vis-NIR spectrophotometers were both comparably useful to predict soil texture in 282 soil samples collected from proportional fields in four Canadian provinces.

## Figures and Tables

**Table 1:** Selected examples from literature comparing quantitative predictions of sand, clay, and SOC using spectral response from vis-NIR and mid-IR spectrophotometers.

Soil Property ^a^	Instrument Type	Method	No. of Sample	No. of PLS Factor	Range	Unit	RMSE	R^2^
Sand [1]	^b^	^d^	116	7	58–84	^g^	2.61	N/A
Sand [14]	^b^	^d^	88	N/A	21–96	^f^	N/A	0.93
Clay [1]	^b^	^d^	116	7	8–24	^g^	1.74	N/A
Clay [9]	^b^	^d^	281	6	N/A	^f^	8.88	0.80
Clay [14]	^b^	^d^	88	N/A	2–49	^f^	N/A	0.86
SOC [1]	^b^	^d^	118	6	0.81–1.98	^g^	0.15	N/A
SOC [9]	^b^	^d^	298	9	N/A	^f^	1.08	0.92
SOC [12]	^b^	^d^	31	N/A	0.68–12.0	^f^	0.63	0.96
SOC [18]	^b^	^d^	201	3	0.11–2.63	^f^	0.18	0.91
SOC [18]	^b^	^d^	201	4	0.11–2.64	^f^	0.18	0.91
SOC [18]	^b^	^e^	201	5	0.11–2.65	^f^	0.21	0.87
SOC [18]	^b^	^e^	201	5	0.11–2.66	^f^	0.29	0.75
Sand [1]	^c^	^d^	116	N/A	58–84	^g^	3.33–3.77	N/A
Sand [4]	^c^	^d^	457	N/A	80–900	^h^	108	0.76
Sand [5]	^c^	^d^	319	N/A	2–71	^g^	7.2	0.80
Sand [7]	^c^	^d^	743	8	1–95	^g^	11.93	0.82
Clay [1]	^c^	^d^	116	N/A	8–24	^g^	1.91–2.28	N/A
Clay [4]	^c^	^d^	457	N/A	50–790	^h^	75	0.78
Clay [5]	^c^	^d^	319	N/A	8–53	^f^	3.8	0.90
Clay [7]	^c^	^d^	743	12	0.7–35	^f^	4	0.67
SOC [1]	^c^	^d^	118	N/A	0.81–1.98	^g^	0.18	N/A
SOC [3]	^c^	^d^	108	6	15–144	^h^	6.2	0.89
SOC [4]	^c^	^d^	674	N/A	2–59	^h^	3.1	0.80
SOC [6]	^c^	^d^	103	6	0.82–8.9	^f^	0.5	0.90

^a^ Reference; ^b^ mid-IR; ^c^ vis-NIR; ^d^ ex situ; ^e^ field moist; ^f^ %; ^g^ dag kg^−1^; ^h^ g kg^−1^.

**Table 2:** Statistical data of soil properties (analyzed using conventional laboratory methods) observed in both the calibration and validation sets.

Dataset Type	Statistic	Sand (g kg^−1^)	Clay (g kg^−1^)	SOC (g kg^−1^)
Calibration	Min	0	42	5.41
	Median	340	280	16.00
	Max	860	740	39.06
	Mean	378	290	17.11
	SD	203	142	5.99
Validation	Min	0	50	9.71
	Median	330	280	15.94
	Max	860	750	37.54
	Mean	370	299	17.64
	SD	236	163	6.14

**Table 3:** Statistical results of PLSR with leave-one-out cross-validation trained on mid-IR calibration dataset examples and tested on mid-IR validation dataset examples.

Attribute (g kg^−1^)	Dataset Type	No. of PLSR Factors	R^2^	RMSE	SDE	ME
Sand	Calibration	5	0.64	121.4	121.7	0.05
	Validation		0.82	103.3	103.2	1.20
Clay	Calibration	4	0.61	88.6	88.8	−0.03
	Validation		0.78	77.9	78.3	−0.25
SOC	Calibration	6	0.63	3.7	3.7	0.00
	Validation		0.54	4.1	4.2	−0.02

**Table 4:** Statistical results of PLSR with leave-one-out cross-validation trained on vis-NIR calibration dataset examples and tested on vis-NIR validation dataset examples.

Attribute (g kg^−1^)	Dataset Type	No. of PLSR Factors	R^2^	RMSE	SDE	ME
Sand	Calibration	15	0.74	104.0	104.0	0.14
	Validation		0.72	127.3	124.8	−2.85
Clay	Calibration	7	0.79	65.7	65.8	−0.02
	Validation		0.82	71.7	71.9	−0.58
SOC	Calibration	12	0.62	3.8	3.8	−0.01
	Validation		0.49	4.5	4.5	0.09

## Data Availability

The data presented in this study are available on request.
